# Sensitivity and specificity of the Bamberg Dementia Screening Test’s (BDST) full and short versions: brief screening instruments for geriatric patients that are suitable for infectious environments

**DOI:** 10.1186/s12916-021-01927-4

**Published:** 2021-03-05

**Authors:** Wolfgang Trapp, Susanne Röder, Andreas Heid, Pia Billman, Susanne Daiber, Göran Hajak

**Affiliations:** 1 Department of Psychiatry, Sozialstiftung Bamberg, St-.Getreu-Straße 18, 96049 Bamberg, Germany; 2 Department of Physiological Psychology, Otto-Friedrich University Bamberg, Markusplatz 3, 96045 Bamberg, Germany; 3 Department of Geriatric Rehabilitation, Sozialstiftung Bamberg, St-.Getreu-Straße 18, 96049 Bamberg, Germany

**Keywords:** Memory, Dementia, Alzheimer’s disease, Diagnostic, Screening test, Sensitivity, Specificity, Geriatrics

## Abstract

**Background:**

Currently, many patients suffering from dementia do not have a diagnosis when admitted to geriatric hospitals. This is the case despite an increased risk of complications affecting the length of stay and outcome. Unfortunately, many dementia screening tests cannot be used on geriatric inpatients, who are often bedridden. Therefore, we aimed at evaluating the diagnostic accuracy of a small battery of bedside tasks that require minimal vision and fine motor skills in patients with suspected dementia.

**Methods:**

In this prospective study, the Bamberg Dementia Screening Test (BDST) was administered to a consecutive series of 1295 patients referred for neuropsychological testing. The diagnosis of dementia was confirmed in 1159 and excluded in 136 patients.

Sensitivity and specificity for the first subtest (ultra-short form), the first two subtests (short form), and the total score of the BDST were obtained via receiver operating characteristic curves and compared with the sensitivity and specificity values of the Mini-Mental Status Examination (MMSE).

**Results:**

The overall diagnostic quality of the BDST was superior to the MMSE for mild Alzheimer’s dementia (sensitivity and specificity = .94 (95% CI .92 to .96) and .82 (95% CI .75 to .88) vs. .79 (95% CI .76 to .83) and .88 (95% CI .82 to .93)) as well as for other subtypes of mild dementia (sensitivity and specificity = .91 (95% CI .88 to .94) and .82 (95% CI .75 to .88) vs. .72 (95% CI .67 to .76) and .88 (95% CI .82 to .93)). Even the short form of the BDST was comparable to the MMSE regarding sensitivity and specificity. For moderate dementia, it was possible to identify dementia cases with sufficient and excellent diagnostic quality by using the ultra-short and the short form.

**Conclusions:**

The BDST is able to detect dementia in geriatric hospital settings. If the adaptive algorithm is used, administration time can be reduced to less than 2 min in most cases. Because no test materials have to be exchanged, this test is particularly suitable for infectious environments where contact between the examiner and the person being tested should be minimized.

**Supplementary Information:**

The online version contains supplementary material available at 10.1186/s12916-021-01927-4.

## Background

### Dementia as a challenge in general hospitals

Dementia, defined by the World Health Organization’s International Classification of Diseases (ICD 10) as a syndrome characterized by a disturbance of multiple higher cortical functions and associated with deterioration in emotional control, social behavior, or motivation, is a major health problem in industrialized countries [1]. At the moment, about 50 million people worldwide [2] are affected. This number is predicted to rise to more than 130 million people worldwide by 2050 [3].

The majority of subjects suffering from symptoms of dementia do not have a dementia diagnosis when admitted to a hospital [4, 5] and a substantial part of these undiagnosed patients remain undiscovered during their stay [6].

Early diagnosis is important and highly recommended by the Alzheimer Cooperative Valuation in Europe [7]. However, less than half of the people in the general population suffering from dementia receive a formal diagnosis [7–9], although this would be important for several reasons: First, interventions to slow down the progression of cognitive deficits could be initiated. Second, care plans could be implemented while patients still have legal capacity and third, institutionalization might be postponed [10]. Furthermore, all these interventions have proven to enhance quality of life and to delay admission to institutional care [11, 12].

Whereas routine screening of older adults for dementia in the community may not make sense [13], it might be advisable for older people in general hospitals, who form a substantial part of the population treated there [14]. Estimates of the prevalence of dementia in elderly patients of general hospitals range from 15 to 42% [14–16], which is a much higher proportion compared to the prevalence rates in the general population. Several lines of evidence suggest identifying these patients as soon as possible after admission, as they are more difficult to care for [15] and have an increased risk of complications like falls, poor nutrition, or hydration [17], which affect their length of stay [16] and their outcome [18].

### Why still another dementia screening test?

The findings presented above provide strong arguments towards performing cognitive screenings for geriatric patients in general hospitals. Several validated screening measures have proven sufficient accuracy ruling in patients with suspected dementia and ruling out patients without any cognitive impairment and are recommended for dementia screening in current guidelines [19–21]. However, these tests place high demands on the elderly being tested. This includes the ability to perform fine motor skills like drawing, writing, or the demonstration of complex gestures and vision, reading of texts, connecting figures by lines, and complex test instructions and materials. All of them significantly hamper their application in geriatric, often bedridden inpatients in general hospitals.

Hence, our goal was to develop a small battery of true bedside tasks that can be administered without any special materials and assure a sensitive and valid assessment of dementia in hospital settings. The Bamberg Dementia Screening Test (BDST) was used as a basis because its tasks do not require the participant to write, read or draw on paper, and require minimal demands regarding vision and fine motor skills. Pilot results regarding the validity and diagnostic accuracy for the BDST battery, obtained from a small sample, had been very encouraging [22]. Our objective in this study was to cross-validate these results and to evaluate the sensitivity and specificity of the full and two shortened versions in a larger sample. Based on these results, our goal was to provide an adaptive mode of administration with shortened administration time (offering an algorithm for “short” and “ultra-short” versions) for patients with mild but also pronounced cognitive impairments.

Our primary goals in this study were

- To examine whether the BDST’s true bedside tasks with minimal demands on vision and fine motor skills and brief application time of about 7 min can detect possible cases of dementia in a hospital setting,
- To examine whether shorter versions of the test could be used to reduce the testing effort for patients and test providers, and
- To develop an application algorithm for BDST versions.

## Methods

### Participants

In this prospective study, a consecutive series of patients who were referred for neuropsychological testing on a routine basis, because of a suspected cognitive decline or due to their wish, were recruited from the departments of geriatric internal medicine and geriatric psychiatry of a general hospital in Bamberg, Germany, between January 2016 and February 2020. All of them underwent routine laboratory screening including thyroid function parameters, lues serology, B12 and folic acid levels, a cranial computer tomography (CT) or magnetic resonance imaging (MRI) scan, EEG, ECG, and a thorough psychiatric, neurological, and physical examination to secure a dementia diagnosis.

The neuropsychological tests and other diagnostic procedures were performed during the same inpatient stay, i.e., within 1 week before or after the index test (BDST, see below). All patients were seen by a senior psychiatrist. The decision as to whether the examined patient had dementia was made at a multidisciplinary meeting using ICD 10 criteria for the diagnosis of dementia as well as additional established criteria [23–29] for the diagnosis of mild cognitive impairment (MCI) and dementia subtypes. Participants diagnosed with mild cognitive impairment and patients exhibiting significant depressive symptoms, which could influence the test results, were excluded.

### Neuropsychological test battery and symptom measures

The German version of the Consortium to Establish a Rationale in Alzheimer‘s Diagnostic neuropsychological battery (CERAD-Plus, [30]) and the Bamberg Dementia Screening Test (BDST [22];) were administered to all participants.

In its current version, the CERAD-Plus battery includes the Mini-Mental State Examination (MMSE [31]; and eleven other tasks covering phonematic fluency, semantic fluency, naming (Boston Naming Test), word list learning, delayed free recall and recognition of a word list, figure drawing (copying geometric shapes), delayed figure recall, and the Trail Making Tests, forms A and B.

Statistical analyses included raw scores for all the CERAD-Plus and BDST subtests as well as total scores for the MMSE and the BDST.

The BDST can be administered in about 7 min and consists of six brief subtests (see Table 1 for cognitive domains, scoring and sample items; domain and total scores are obtained by simply adding the scores for the corresponding items). No test materials are needed and the participants do not have to read, write, draw on paper, or perform complex gestures. Test forms including instructions in English and German are included in the supplemental material (Additional file 1: BDST test form in English (PDF), Additional file 2: BDST test form in German (PDF)).

**Table 1:** Cognitive domains, scoring and sample items of the BDST

Cognitive domain	Task and sample item
Semantic memory *(5 items, maximum score: 10 points)*	**Items 1 to 4** Verbal descriptions of animals are given (e.g., “*What is the name of the animal with the very long neck*”). If the correct name is given (“*giraffe*”), 2 points are awarded. Otherwise, an additional hint is given (e.g., “*The animal lives in Africa and has a yellow –brownish pattern*”). If the answer is now correct, 1 point is awarded. **Item 5** serves as distractor item: *“Are people more afraid of a lion or a rabbit?”* If the answer is “lion,” 1 point is scored and an additional question is asked: *“Why should you be more afraid of a lion?”* For a correct answer *(“dangerous,” “carnivore,”* etc.*, not: “bigger”),* the participant receives another point.
Verbal memory *(4 items, maximum score: 8 points)*	**Free recall** *“What animals did I ask you about before we talked about the lion and the rabbit?”* For each animal that was remembered correctly, 2 points are given. **Recognition** For each animal that could not be remembered, the participant is allowed to choose between three options *(“Did I ask about a leopard, a giraffe or a parrot?”).* If the correct animal is selected, 1 point is awarded.
Visual construction *(4 items, maximum score: 8 points)*	The participant is asked to draw shapes with the index finger. “*Please watch carefully* [administrator draws a symbol with her/his index finger] *and then try to draw the following shapes in the air.*” Example: If the shape is drawn correctly by the participant (shape can be clearly recognized, regardless if a mirror image is drawn or not) 2 points are awarded. If not, the shape is repeated by the administrator (*“I’ll draw the shape again …*. *Please try again now”).* If the shape is now drawn correctly, 1 point is awarded.
Verbal fluency *(1 item, maximum score: 8 points)*	Naming of larger cities (≥ 50,000 inhabitants) anywhere in the world in 60 s. 1 point is awarded for each 3 cities (8 points for 24 or more cities).
Visual memory *(4 items, maximum score: 8 points)*	**Free recall** *“What figures did we draw in the air before we talked about big cities?”* For each shape that was remembered correctly, 2 points are given. **Recognition** For the remaining shapes that could not be remembered, hints are given *(“one shape looked like a letter”)*. If the correct shape is drawn, one point is awarded.
Cognitive flexibility *(4 items, maximum score: 8 points)*	The participant is asked to reproduce tapping patterns given by the administrator *(“Please watch carefully and then try tapping the same pattern”*). If the pattern is reproduced correctly, 2 points are awarded. If not, the pattern is given again by the administrator (*“I’ll tap the pattern again …. Please try again now”).* If the pattern is now reproduced correctly, 1 point is awarded.

To evaluate under which circumstances a shortened version of the BDST would be sufficient, the BDST total sum score, the score for the first subtest (“ultra-short form,” denoted as BDST_us_ in the following text), and the sum score for the first and second subtest (“short form,” denoted as BDST_s_ in the following text) were used for further analyses. The two tests were given within the same test session.

The tests were performed by seven neuropsychologists (S.R., A.H., P.B., W. T and the three colleagues we have thanked in the “Acknowledgments” section) with several years of experience in performing relevant diagnostic tests in people with dementia. All of them were trained to perform the BDST by the first author.

The CERAD-Plus but not the BDST results were available to the senior psychiatrists or senior internists and their multidisciplinary team. Additionally, the MMSE scores—following the German Guideline for dementia [19]—were considered to distinguish between mild and moderate forms of dementia. Thus, the BDST results were not available to the persons who constructed the reference standard.

Furthermore, all patients completed the German short version of the Geriatric Depression Scale (GDS) [32], a brief screening instrument for depressive symptoms in the elderly. Participants with GDS scores higher than 5, indicating possible depression, were excluded.

CERAD-Plus, BDST, and GDS were administered by psychologists of the geriatric or psychiatric ward.

### Statistical analyses

#### Comparability of the three diagnostic groups

Univariate analyses of variance with Scheffé a posteriori analyses were performed to compare age, GDS scores, and years of education in the four diagnostic groups (CNT, DEM_mi_alz_, DEM_mi_other_, and DEM_mo_) and a chi-square test was used to test for comparison of the male to female ratio across these groups.

#### Validity and diagnostic quality of the BDST

To gather information about the BDST’s concurrent validity, Pearson correlation coefficients were computed between BDST and CERAD-Plus subtasks and total scores. Following the procedure suggested by Schmidt et al. [33], a CERAD “composite” value was obtained by extracting one principal component from the highly intercorrelated CERAD-Plus subtests to obtain one summative CERAD score that can be correlated with the BDST and MMSE total scores.

Univariate analyses of variance with Scheffé a posteriori analyses were performed to compare the diagnostic groups’ (CNT, DEM_mi_alz_, DEM_mi_nonalz_, and DEM_mo_) BDST scores.

MMSE, BDST total, BDST_s_, and BDST_us_ scores were used to plot receiver operating characteristic (ROC) curves of sensitivity against 1-specificity. This was done

- Using MMSE, BDST total, and BDST_s_ scores for mild dementia vs. control subjects, for DEM_mi_alz_ vs. CNT and DEM_mi_other_ vs. CNT,
- Using BDST total, BDST_s_, and BDST_us_ scores for moderate dementia vs. control subjects, and
- Using MMSE, BDST total, and BDST_s_ scores for mild or moderate dementia vs. control subjects

The different ROC curves were compared using the pROC – package [34] of R [35], which utilizes the formula provided by [36] for paired ROC curves. The pROC – package also computes tests for unpaired ROC curves where the *p* value is computed with an unpaired *t*-test with unequal sample size and unequal variance.

Optimum cutoff scores were determined using the Youden index. Sensitivity and specificity were computed for all screening measures based on the cutoff scores found.

Sensitivity (“How many persons that are suffering from dementia are detected by a screening test?”) and specificity (“How many persons showing no cognitive deficits are correctly categorized as unimpaired by the screening?”) refer to a situation where the “true diagnosis” (for example “mild dementia” vs. “cognitively unimpaired”) is known.

Unfortunately, this is not the case in practice, where the “true diagnosis” has to be inferred from the result of a screening test. The positive predictive value specifies to what extent a screening test indicating a diagnosis (in our case for example: “mild dementia”) is right. Even more importantly, the negative predictive value answers the question to what extent an inconspicuous test result, which prevents further diagnostic or protective action (for example to protect dementia patients in a geriatric ward), is correct. Although positive and negative predictive values depend on sensitivity and specificity, they also vary with the prevalence of the diagnostic condition (i.e., the percentage of people suffering from dementia) in a specific setting. Therefore, curves of negative predictive values against the prevalence of dementia are plotted based on the sensitivities and specificities found in the ROC analyses described above.

#### Effects of age, gender, and years of education on BDST performance

BDST scores were correlated with age, gender, and years of education using Pearson correlation coefficients.

To estimate to what extent the BDST scores are influenced by age, gender, and years of education independently from cognitive achievement, three stepwise linear regressions were performed using the BDST total, BDST_s_, or BDST_us_ scores as dependent variable and all CERAD-Plus scores as well as gender, age, and years of education as predictors in order to examine whether age, gender, and years of education or cognitive achievement primarily influence test performance.

The significance level was set to *α* < 0.05, two-tailed, for all analyses.

## Results

### Sample characteristics

In total, 1905 potentially eligible patients were referred for neuropsychological testing. Seventy two of them were discharged before testing or a thorough diagnosis could be performed and 23 did not agree to participate. For 20 patients, other causes of cognitive decline were identified (three patients were found to have had a stroke and 17 participants suffered from Wernicke-Korsakoff syndrome caused by alcohol abuse). In total, 292 participants were excluded because of significant depressive symptoms and 186 patients because of a diagnosis of MCI. Seventeen participants were unable to perform the neuropsychological test battery because of severe hearing or visual impairments.

Of the remaining 1295 participants, 883 met diagnostic criteria for mild dementia. A total of 519 of these were suffering from Alzheimer’s disease (DEM_mi_alz_) and the remaining 364 received a dementia diagnosis other than Alzheimer’s (DEM_mi_other_, 19.0% vascular disease, 19.6% frontotemporal disease, 1.9% Parkinson’s disease dementia, and 0.7% Levy Body dementia). In total, 276 participants met criteria for moderate dementia (DEM_mo_). Then, 136 showed no signs of cognitive impairment and were thus included as clinical control sample (CNT).

No significant differences were found between the four groups concerning age (F_(3,1291)_ = 1.029, *p* = .379), GDS scores (F_(3,1291)_ = .606, *p* = .611), years of education (F_(3,1291)_ = 1.100, *p* = .348), and gender (χ^2^_(3)_ = 4.089, *p* = .252), see also Table 2 for more detailed information about the sample.

**Table 2:** Sample characteristics. *SD* standard deviation, *CNT* clinical control sample, *DEM*_*mi*_ mild dementia, *DEM*_*mi_alz*_ mild dementia, Alzheimer’s disease, *DEM*_*mi_other*_ mild dementia, other forms, *DEM*_*mo*_ moderate dementia, *TMT A* Trail Making Test Form A, *TMT B* Trail Making Test Form B

	CNT (***n*** = 136)	DEM_**mi**_ (***n*** = 883)	DEM_**mi_alz**_ (***n*** = 519)	DEM_**mi_other**_ (***n*** = 364)	DEM_**mo**_ (***n*** = 276)
**Gender**	*female/male*	*female/male*	*female/male*	*female/male*	*female/male*
	80 / 56	544 / 339	332 / 187	212 / 152	161 / 115
	*mean (SD)*	*mean (SD)*	*mean (SD)*	*mean (SD)*	*mean (SD)*
**Age**	75.64 (7.82)	76.34 (8.45)	76.67 (8.50)	75.87 (8.41)	76.33 (6.43)
**Years of education**	12.13 (2.02)	12.04 (1.94)	12.01 (1.93)	12.09 (1.97)	11.84 (1.75)
**GDS**	3.75 (3.29)	3.74 (3.29)	3.66 (3.45)	3.83 (3.07)	3.45 (3.12)
**BDST**					
**Total score**	41.29 (4.06)	29.61 (5.87)	29.21 (5.66)	30.16 (6.11)	19.66 (5.40)
**Short form**	16.81 (1.13)	14.00 (2.75)	13.62 (2.75)	14.54 (2.67)	9.96 (3.61)
**Semantic memory (=ultra-short form)**	9.76 (.62)	8.81 (1.62)	8.78 (1.61)	8.85 (1.64)	6.72 (2.46)
**Verbal memory**	7.04 (.86)	5.19 (1.75)	4.84 (1.81)	5.70 (1.54)	3.24 (1.99)
**Visual construction**	7.67 (.59)	6.41 (1.69)	6.52 (1.58)	6.25 (1.82)	4.76 (2.15)
**Verbal Fluency**	4.33 (3.16)	2.05 (1.86)	2.00 (1.88)	2.12 (1.84)	1.07 (1.25)
**Visual memory**	6.99 (1.16)	3.43 (2.08)	3.15 (1.96)	3.84 (2.19)	1.38 (1.45)
**Cognitive flexibility**	5.42 (1.56)	3.63 (1.38)	3.78 (1.45)	3.42 (1.25)	2.75 (1.14)
**MMSE**					
**Total score**	28.14 (1.79)	24.09 (3.02)	23.78 (2.88)	24.53 (3.16)	15.80 (3.15)
**CERAD-Plus**					
**Semantic fluency**	20.38 (5.41)	12.06 (4.25)	12.03 (4.40)	12.10 (4.04)	7.53 (3.57)
**Boston naming test**	14.18 (1.08)	12.06 (2.21)	12.10 (2.18)	12.00 (2.25)	10.14 (2.60)
W**ord list** **learning**	18.82 (2.97)	11.71 (3.88)	10.76 (3.63)	13.05 (3.83)	7.94 (3.93)
**Word list delayed free recall**	6.21 (1.61)	2.55 (2.00)	1.65 (1.55)	3.83 (1.86)	1.11 (1.55)
**Percent word list recognition**	96.36 (8.20)	83.46 (13.50)	78.67 (13.79)	90.24 (9.64)	71.18 (14.09)
**Figure drawing**	10.61 (.79)	8.83 (1.879	8.94 (1.82)	8.67 (1.93)	7.05 (2.48)
**Delayed recall figures**	8.43 (2.43)	3.09 (2.75)	2.42 (2.57)	4.04 (2.73)	1.01 (1.96)
**TMT A**	51.83 (18.40)	102.95 (45.1)	98.63 (45.29)	108.78 (44.03)	156.65 (37.33)
**TMT B**	135.17 (52.97)	268.96 (57.30)	262.37 (63.96)	277.79 (45.52)	299.71 (2.74)
**Phonematic fluency**	11.80 (4.15)	6.90 (3.74)	7.19 (3.74)	6.49 (3.70)	5.11 (3.45)

### Validity and diagnostic accuracy

Table 3 shows correlation coefficients between all BDST and selected CERAD-Plus subtests of corresponding content. Subtests covering similar contents yielded meaningful correlations between each other. Remarkably, even conceptually similar subtests differing remarkably in their mode of presentation (recognition of tapping patterns vs. connecting of letters and figures, recall of simple shapes drawn in the air vs. recall of geometric shapes drawn on paper) were substantially correlated.

**Table 3:** Correlations between BDST and CERAD-Plus scores corresponding in content (*p* < .0005 for all coefficients, TMT B: Trail Making Test B)

BDST subtest	corresponding CERAD-Plus subtests		
*Semantic memory*	***Boston naming test***		***Semantic fluency***
	.54		.44
*Verbal memory*	***Word list***		
	*Learning*	*Delayed free recall*	*Recognition*
	.47	.49	.41
*Visual construction*	***Figure drawing***		
	.42		
*Verbal fluency*	***Fluency***		***TMT B***
	*Semantic*	*Phonematic*	
	.68	.54	−.56
*Visual memory*	***Delayed recall figures***		
	.57		
*Cognitive flexibility*	***TMT B***		
	−.52		

High correlations were found for the BDST total score and the MMSE and CERAD-Plus total scores (*r* = .71 and *r* = .77 respectively, *p* < .0005 each). Even the shortened versions of the BDST (BDST_s_, *r* = .60 and *r* = .56 and BDST_us_, *r* = .49 and *r* = .42, *p* < .0005 for all coefficients) are still moderately related to the MMSE and CERAD-Plus total scores.

**Fig. 1:**
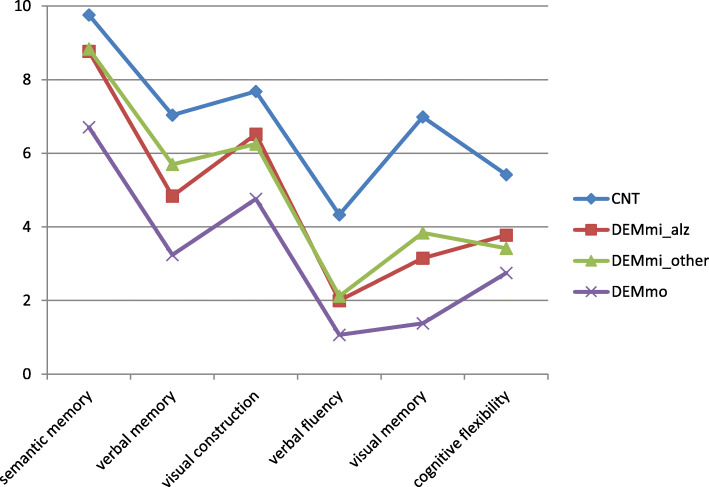
Average scores for the BDST subtests (see also Table 1 for scoring rules) for patients suffering from mild Alzheimer’s disease (DEM_mi_alz_), mild dementia of other types (DEM_mi_other_) or moderate dementia (DEM_mo_) and cognitively unimpaired participants (CNT)

All BDST subtest scores, the BDST total score and the two shortened versions of the BDST differentiated between the four groups (F_(3,1291)_ between 274.742 and 86.810, *p* < .0005 each, see also Fig. 1).

Scheffé a posteriori comparisons indicate that all BDST scores were different for control subjects, patients with mild and patients with moderate dementia (*p* < .0005 for each comparison). Additionally, DEM_mi_alz_ patients scored lower than DEM_mi_other_ patients in the verbal and visual memory subtests (*p* < .0005 each), whereas DEM_mi_other_ patients scored lower than DEM_mi_alz_ patients in the cognitive flexibility task of the BDST (*p* = .002).

Table 4 shows the results of the ROC analyses regarding the diagnostic accuracy of the MMSE and BDST scores for the diagnosis of mild and moderate dementia.

**Table 4:** Diagnostic accuracy of the BDST and the MMSE (95% confidence intervals in parentheses). *SEN* sensitivity, *SPE* specificity, *AUC* area under the curve, *Dem*_*mo*_ moderate dementia, *Dem*_*mi*_ mild dementia, *Dem*_*mi_alz*_ mild dementia Alzheimer’s disease, *Dem*_*mi_other*_ mild dementia, other types than Alzheimer’s disease, *BDST* Bamberg Dementia Screening Test, *BDST*_*s*_ Bamberg Dementia Screening Test, short form (sum score for the first and second subtest), *BDST*_*us*_ Bamberg Dementia Screening Test, ultra-short form (only score for the first subtest), *MMST* Mini-Mental State Examination. (1) Following the German Guideline for dementia, the MMSE scores were considered to distinguish between mild and moderate forms of dementia. Therefore, no measures of diagnostic accuracy can be presented for the MMSE and moderate dementia

		Dem_mo_ vs. CNT	Dem_mi_ vs. CNT	Dem_mi_alz_ vs. CNT	Dem_mi_other_ vs. CNT	Dem_mi_ or Dem_mo_ vs. CNT
MMSE	Cut-off	(1)	≤ 26/30	≤ 26/30	≤ 26/30	≤ 26/30
	SEN		.76 (.73–.79)	.79 (.76–.83)	.72 (.67–.76)	.81 (.79–.83)
	SPE		.88 (.82–.93)	.88 (.82–.93)	.88 (.82–.93)	.88 (.82–.93)
	NPV		.36 (.31–.41)	.52 (.46–.59)	.54 (.47–.60)	.35 (.30–.40)
	PPV		.98 (.96–.99)	.96 (.94–.98)	.94 (.91–.97)	.98 (.97–.99)
	AUC		.88 (.86–.91)	.90 (.88–.92)	.86 (.83–.88)	.91 (.89–.93)
BDST	Cut-off	≤ 31/50	≤ 37/50	≤ 37/50	≤ 37/50	≤ 37/50
	SEN	1.000	.93 (.91–.94)	.94 (.92–.96)	.91 (.88–.94)	.94 (.93–.95)
	SPE	1.000	.82 (.75–.88)	.82 (.75–.88)	.82 (.75–.88)	.82 (.75–.88)
	NPV		.63 (.56–.70)	.77 (.70–.84)	.77 (.70–.84)	.62 (.54–.69)
	PPV		.97 (.96–.98)	.95 (.93–.97)	.93 (.90–.96)	.98 (.97–.99)
	AUC	1.000	.95 (.94–.97)	.96 (.95–.97)	.94 (.92–.96)	.96 (.95–.97)
BDST_s_	Cut-off	≤ 15/18	≤ 16/18	≤ 16/18	≤ 16/18	≤ 16/18
	SEN	.94 (.91–.96)	.84 (.81–.86)	.87 (.85–.90)	.79 (.75–.83)	.86 (.84–.88)
	SPE	.93 (.89–.97)	.70 (.62–.77)	.70 (.62–.77)	.70 (.62–.77)	.70 (.62–.77)
	NPV	.88 (.82–.93)	.40 (.34–.46)	.59 (.52–.67)	.55 (.48–.62)	.26 (.22–.30)
	PPV	.97 (.94–.99)	.95 (.93–.96)	.92 (.89–.94)	.87 (.84–.91)	.99 (.98–.99)
	AUC	.97 (.95–.98)	.86 (.83–.88)	.89 (.86–.91)	.81 (.78–.85)	.88 (.86–.90)
BDST_us_	Cut-off	≤ 9/10	–	–	–	≤ 9/10
	SEN	.85 (.81–.89)				.58 (.55–.61)
	SPE	.86 (.81–.92)				.85 (.79–.91)
	NPV	.74 (.67–.81)				.20 (.16–.23)
	PPV	.93 (.89–.96)				.97 (.96–.98)
	AUC	.90 (.87–.93)				.73 (.70–.77)

The characteristics show that both MMSE and BDST are slightly better at detecting patients suffering from mild Alzheimer’s disease than patients suffering from other mild forms of dementia. The areas under the curve (AUC) indicate that the overall diagnostic quality of the full version of the BDST is superior to the MMSE. The full version of the BDST outperforms the MMSE for all participants with mild dementia (*z* = 4.784, *p* < .0005), as well as the two subsamples of participants with Alzheimer’s disease (*z* = 3.927, *p* < .0005) and other forms of dementia (*z* = 4.706, *p* < .0005). Similar results are obtained when all patients with mild or moderate dementia are taken into account (right column of Table 4, *z* = 4.619, *p* < .0005). The AUCs for the short form of the BDST and the MMSE do not differ significantly when patients with mild dementia are considered (*z* = 1.665, *p* = .096 for all, *z* = 1.015, *p* = .310 for Alzheimer’s disease and *z* = 1.949, *p* = .051 for other forms), or when all patients with mild or moderate dementia are taken into account (*z* = 1.949, *p* = .051).

Whereas the overall diagnostic quality of the MMSE (*z* = 2.101, *p* = .036) and the BDST_s_ (*z* = 3.110, *p* = .002) is higher for patients with mild Alzheimer’s disease than for patients with other mild forms of dementia, no such difference for the long form of the BDST could be found (*z* = 1.395, *p* = .163).

For moderate dementia, even the first subtest of the BDST (BDST_us_ score) seems to be sufficient to detect dementia with reasonable sensitivity and specificity, while the first two subtests (BDST_s_ score) do so with excellent diagnostic quality.

**Fig. 2:**
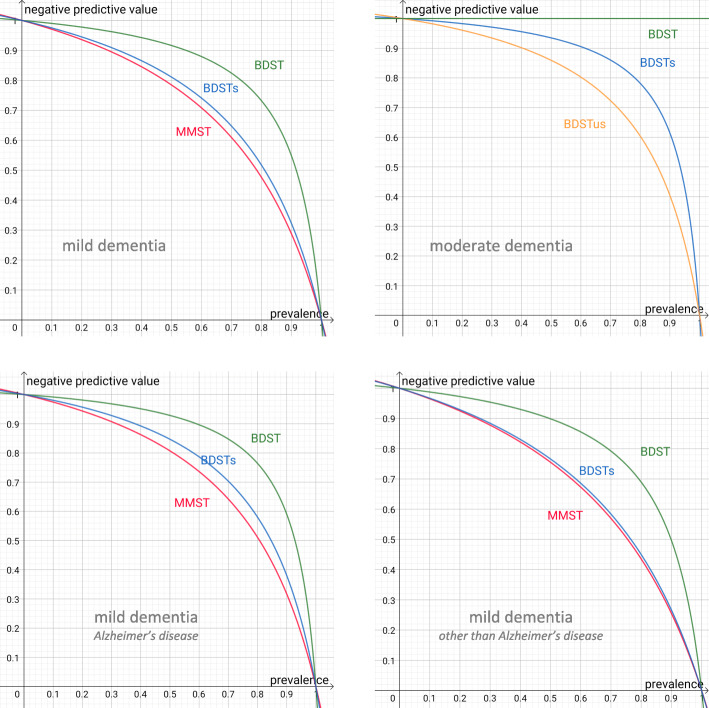
Calculated negative predictive values depending on the prevalence rate for dementia based on the sensitivities and specificities for the MMSE and different forms of the BDST shown in Table 4. BDST: Bamberg Dementia Screening Test, BDST_s_: Bamberg Dementia Screening Test, short form (sum score for the first and second subtest), BDST_us_: Bamberg Dementia Screening Test, ultra-short form (only score for the first subtest), MMST: Mini-Mental State Examination. Negative predictive values (no further action indicated according to the test result) computed based on the for prevalence estimates

In Fig. 2, the negative predictive values (no further action indicated according to the test result) are plotted against the estimated prevalence of dementia. It can be seen that the BDST shows excellent negative predictive values superior to the MMSE, even for populations with up to 70% of dementia cases. Again, both measures’ values are marginally higher for Alzheimer’s disease than for other types of dementia. Even the shortened version of the BDST (BDST_s_) shows slightly better negative predictive values than the MMSE. For cases with moderate dementia, the brief version (BDST_s_) might be sufficient in many cases. Even when the very brief BDST_us_ score is used, only few patients remain unidentified in the majority of scenarios.

### Effects of age, gender, and education on BDST test scores

Only minor correlations of age, gender, and years of education could be found with the three BDST scores (*r* = −.20 to .10, see also Additional file 3: Correlations of the BDST scores with age, gender, and years of education).

When years of education, gender, and age together with all CERAD-Plus subscores including the MMSE are entered as predictors in a stepwise linear regression using the three BDST scores as dependent variable, only the CERAD-Plus scores remain as significant predictors (BDST: MMSE (beta = .277), Boston Naming Test (beta = .198), delayed recall figures (beta = .165), semantic fluency (beta = .156), TMT B (beta = −.119), word list learning (beta = .125), and figure drawing (beta = .074) *p* < .0005 each except figure drawing: *p* = .002; BDST_s_: MMSE (beta = .238), Boston Naming Test (beta = .294), word list learning (beta = .120), semantic fluency (beta = .140), word list recognition (beta = .106), figure drawing (beta = −.090), and TMT B (beta = −.059), *p* < .0005 each except figure drawing: *p* = .001 and TMT B: *p* = .031, BDST_us_: Boston Naming Test (beta = .413), MMSE (beta = .159), and semantic fluency (beta = .138) *p* < .0005 each).

## Discussion

In a large sample of 1295 participants, it could be shown that the BDST shows high sensitivity and sufficient specificity regarding the detection of mild dementia. Thereby, the BDST cutoff score of ≤ 37/50 for mild dementia obtained in a smaller sample of a previous study [22] could be cross-validated.

The full version of the BDST proved superior to the MMSE in detecting dementia. This can be ascribed mainly to higher sensitivity values. For instance, the sensitivity of the MMSE regarding mild forms of dementia is .76, indicating that roughly a quarter of patients might remain undetected, while the corresponding sensitivity value for the BDST of .93 is significantly higher.

Even a shortened BDST version consisting of only the first two subtests was shown to be comparable concerning diagnostic quality while surpassing the MMSE with respect to brevity (16 vs. 30 items), the necessity of test materials (no additional test materials needed vs. pencil, watch and two extra sheets of paper needed) and testing time (less than two minutes vs. seven to eight minutes). Notably, the first subtest, requiring less than 1 min of administration time, already proved to detect possible moderate dementia with sufficient sensitivity and specificity.

Based on the sensitivity and specificity values found in this study, high negative predictive values would be expected even if the proportion of dementia cases were up to 40% in the population screened with the BDST.

Table 4 suggests that the BDST_s_ has higher sensitivity, but lower specificity than the MMSE, meaning it leads to more false-positive cases. While the PPVs in Table 4 are excellent (which may be a result of its high sensitivity but surely is also due to the fact that there are many more dementia cases than CNT cases in our sample), the NPVs, like for the MMSE, are not satisfactory. Therefore, the BDST_s_ should be used as a first-stage screening tool. In case the BDST_s_ is positive, patients can be referred to a specialist for a more thorough diagnosis. On the other hand, if the BDST_s_ is negative, the remaining subtests of the BDST should also be administered, at least in settings where many dementia cases are to be expected (see also Fig. 2 on this topic).

It can be argued that by lowering cutoffs, a better balance between SN and SP could be obtained. However, lowering the cutoff to ≤ 36/50 decreases sensitivity from .93 to .88 and increases specificity from .82 to .85, which would lead to an overall worse diagnostic accuracy. We also placed more emphasis on a high detection rate (despite risking a higher false-positive rate), so we decided to keep the cutoff at ≤ 37/50.

**Fig. 3:**
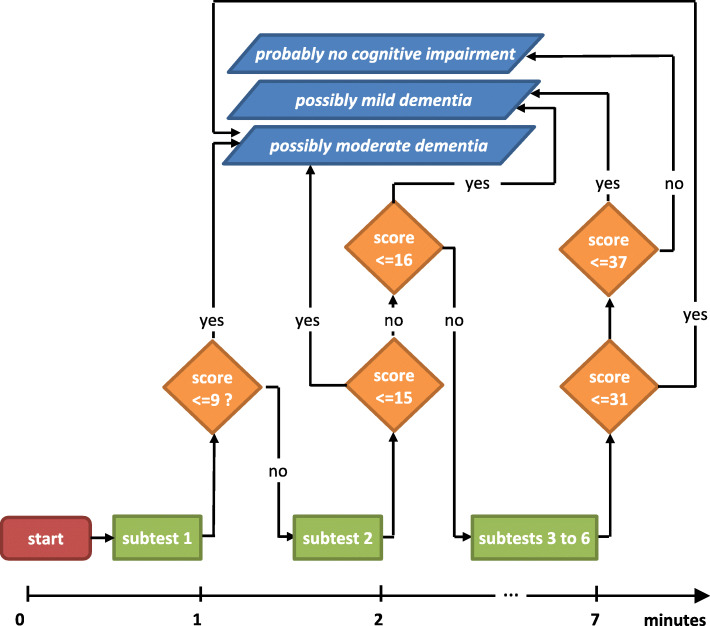
Algorithm for an adaptive application of the BDST

These results justify an adaptive algorithm for application as illustrated in Fig. 3. Any mistake in the first subtest (naming of animals) indicates moderate dementia so that the testing can be terminated, and the patient should be referred to psychiatrists and neuropsychologists.

Otherwise, if in the second subtest there is more than one error in the recall of the animals named in the first subtests (for example two animals could only be recognized but not freely recalled), either mild or moderate dementia can be suspected and the test session could also be terminated at this point.

Nevertheless, as the diagnostic quality of the BDST is higher than that of the BDST_s_, the full BDST should be administered whenever possible. However, if there is too little time to perform the complete test, taking 1 or 2 min for the abbreviated versions has proven to be better than performing no screening at all. However, especially in settings with high prevalence of true positive cases, a positive BDST_s_ score is highly likely to be truly positive, but a negative BDST_s_ does not mean very much. As NPVs are low, clinicians should be cautious about over-interpreting a negative test of the BDST_s_, especially if the score is close to the cutoff point.

The BDST was also able to prove sufficient sensitivity and specificity not only for Alzheimer’s but also for other types of dementia. Notably, different test profiles matching theoretical expectations for participants suffering from either Alzheimer’s or other types of dementia became apparent. As expected, the first subgroup performed worse in the subtests affecting verbal and visual memory while the latter group showed more pronounced deficits in the subtest covering cognitive flexibility. However, as can be seen in Fig. 1, the numerical differences in the scores are very small, and it should be noted that for the other three subtests, no significant differences could be found, which might be caused by the heterogeneity of the non-Alzheimer group. It was not our purpose, though, to develop a screening that can distinguish between Alzheimer’s disease and other forms of dementia but to analyze whether the BDST can detect people with dementia, even when they do not suffer from Alzheimer’s disease.

The total scores of the BDST, MMSE, and CERAD-Plus were highly intercorrelated. This was also true for the scores of CERAD-Plus and BDST subtests with similar content. This is remarkable because of the considerably different modes of administration and test behavior that make the BDST administrable even for bedridden patients. These findings provide additional evidence regarding construct validity of the screening measure introduced in this paper.

Another strength of the test is that—although cognitively healthy control patients experienced virtually no difficulties with the completion of the tasks—no ceiling effects for the BDST occurred. The average BDST score was more than two standard deviations below the BDST’s maximum score, whereas the average MMSE score obtained in our sample was only about one standard deviation below MMSE’s maximum score. Furthermore, no floor effects for subjects suffering from moderate dementia could be detected, as these participants still reached an average score of 20. Thus, the BDST allows for testing a wide range of performance levels of both weakly and strongly impaired individuals, which makes this test particularly suitable for geriatric patients. Furthermore, administration of the BDST does not require investigators to ask considerably basic questions about spatial and temporal orientation, which might be experienced as humiliating and stressful [37].

Also, educational level, gender, or age have shown no substantial effects on test performance, independently of cognitive status, which itself is affected by educational level and age.

It should not go unmentioned that the advantages of the BDST over the MMSE also apply to many alternative tests for dementia and cognitive impairment like the Montreal Cognitive Assessment (MoCA, [38]), the Addenbrooke’s Cognitive Examination III (ACE-III, [39]) or the Test Your Memory (TYM [40]), which have also been shown to outperform the MMSE and to have similar or even better sensitivity and specificity values. The BDST is shorter than most other screenings and can be administered even in cases where conventional screenings cannot be used. For example, if there is minimal time to administer a screening, the TYM, which has excellent diagnostic quality [41], could be a better choice, because, as patients are to fill in the test themselves, administration time is minimized. However, participants must be able to read, write, and draw into relatively small gaps in the test form. For example, a clock face must be drawn into a circle with a diameter of about 3.5 cm (1.2 in). For participants that are bedridden and too physically ill to meet these requirements, the short and full versions of the BDST could therefore be a viable alternative to the TYM.

It should be noted, however, that poor test performance in cognition tests per se could be due to other reasons than dementia, for example delirium, diabetes, depression, or pain [42]. Hence, prior to using cognitive screening tests, these conditions should be excluded.

Generally, neither the BDST nor any other dementia screening test can replace detailed neuropsychological testing and other diagnostic procedures such as cranial CT or MRI scans, and others. Especially in populations where dementia cases are rare, even screening tests with good sensitivity and specificity have low positive predictive values, i.e., subjects who in reality do not have dementia are to be expected to have falsely positive test results. This is why many authors argue that in clinical settings routine dementia screening is not feasible [43]. To overcome this issue, we recommend that dementia screenings should be combined with a quick and valid screening for delirium, e.g., the 4AT [44], and that the term “dementia” should be avoided until careful diagnostics have been carried out.

It should be pointed out that the data obtained for the BDST in this study and presented in this paper was drawn from a clinical sample of patients referred for neuropsychological testing. Although this might be a valid setting for the BDST in many cases (e.g., in a geriatric ward, a quick assessment might be very helpful), this has led to a high proportion of participants with the target condition (dementia), which possibly affected the PPV and NPV estimates.

Furthermore, by selecting the positivity cutoff after performing the test using ROC analyses, we have increased the risk of too optimistic accuracy estimates. However, it should be noted that this effect is strongest in smaller samples. As can be seen in Table 4, for the short and the long version of the BDST, the determined cutoff scores for mild dementia stay the same, regardless of whether the whole sample of participants with dementia or the two subsamples of participants with Alzheimer’s and other forms of dementia are considered.

Although it should be noted that the cutoff score of ≤ 37/50 is a replication of the cutoff score that was determined for a different, albeit smaller sample in a former study [22], our findings have to be cross-validated, desirably in a population-based sample. Furthermore, the development of parallel versions of the BDST would aid in the long-term evaluation of its usefulness in measuring cognitive decline in the course of illness.

When considering the clinical benefits of the BDST test, one has to realize that the identification rate of dementia reporting in hospital records ranges between 26 and 70% in European and North American countries [45–48], which means that many patients suffering from dementia are overlooked. As no prior preparation or test materials are needed and the BDST is easy to administer and evaluate, this test might help to reduce the barriers of using neuropsychological screening tests in geriatric wards of general hospitals or residents of nursing homes. In the optional BDST smartphone app, which can be obtained from the corresponding author, the user is guided through administration, automated scoring, and interpretation of the results and provided with an automatic suggestion for a medical report text. This could help to avoid potential adverse events like falls or poor hydration, which are more likely to occur in patients suffering from dementia and affect their outcome, for example with regard to institutionalization, mortality, and length of stay [49]. Thus, screening measures like the BDST could help to improve outcome and reduce costs of stays in geriatric wards of general hospitals. The fact that no test utensils (pencils, test forms, written instructions, etc.) have to be exchanged between the examiner and the person being tested makes the BDST especially suitable for dementia screenings in infectious environments or remote telemedical testing via video calls. Thus, in rural regions, where no specialized personal is available, a more timely diagnosis of dementia could be initiated.

## Conclusions

In summary, the data presented here yields evidence that “true bedside” measures like the BDST may qualify as valid screening measures for the diagnosis of dementia. Especially in geriatric settings, such measures might help to avoid unwanted effects on the health of overlooked dementia patients with minimal effort.

## Supplementary Information

**Additional file 1.** BDST test form in English.

**Additional file 2.** BDST test form in German.

**Additional file 3.** Correlations of the BDST scores with age, gender and years of education.

## Data Availability

The datasets used and/or analyzed during the current study and the APK file to install the BDST app on Android® devices are available from the corresponding author on reasonable request.

## Abbreviations

4AT: 4 ‘A’s test for detecting delirium
AUC: Area under the curve
BDST: Bamberg Dementia Screening Test
BDST_s_: Bamberg Dementia Screening Test, short form (sum score for the first and second subtest)
BDST_us_: Bamberg Dementia Screening Test, ultra-short form (only first subtest)
CERAD: Consortium to Establish a Rationale in Alzheimer‘s Diagnostic
CNT: Clinical control sample
CT: Cranial computer tomography
DEM_mi_alz_: Mild dementia, Alzheimer’s disease
DEM_mi_other_: Mild dementia, other subtypes
DEM_mo_: Moderate dementia
GDS: Geriatric Depression Scale
ICD: International Classification of Diseases
MMSE: Mini-Mental Status Examination
MRI: Magnetic resonance imaging
ROC: Receiver operating characteristic
SD: Standard deviation
