# Supplementary material for: Sensitivity and specificity of the Bamberg Dementia Screening Test’s (BDST) full and short versions: brief screening instruments for geriatric patients that are suitable for infectious environments
Source: BMC Med. 2021 Mar 5;19:65. doi: 10.1186/s12916-021-01927-4 (PMC7934397; doi:10.1186/s12916-021-01927-4)
Supplement: Supplementary file 1 — Additional file 1. BDST test form in English. [file 12916_2021_1927_MOESM1_ESM.pdf]

# BDST

| ***First, I’m going to ask a few questions about animals.*** | **Points**<br>**Pr.** | **recall** | ***6. What animals did I ask you about before we talked about the lion and the rabbit? (only rate the first 6 answers)*** |
|---|---|---|---|
| *1. What is the name of the animal with the long trunk?*<br>*It’s a very big animal and it can „trumpet“ using its trunk.* | | | *Did I ask about a hippo, an elephant or a rhino?* |
| *2. What is the name of the animal with the very long neck?*<br>*This animal lives in Africa and has a yellow-brownish pattern.* | | | *Did I ask about a leopard, a giraffe or a parrot?* |
| *3. What is the name of the animal which gives its name to a crosswalk with white and black stripes?*<br>*It is an animal that looks like a striped horse.* | | | *Did I ask about a zebra, a tiger or a monkey?* |
| *4. What is the name of the bear that lives in very cold regions with ice?*<br>*Simply put together the words "bear" and "polar" in the correct order.* | | | *Did I ask about a penguin, a seal or a polar bear?* |
| *5. Which animal are people more afraid of: a lion or a rabbit?*<br>*Why?* (dangerous, carnivore etc.) | | | |
| ***Please watch carefully [administrator draws a symbol with her/his index finger] and then try to draw the following shapes in the air***<br>*(award 2 points if the first attempt and 1 point if the second attempt is correct)* | | | ***12. What figures did we draw in the air before we talked about big cities?*** |
| 7. 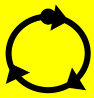 | | | *One shape was round.* |
| 8. 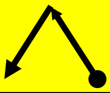 | | | *One shape looked like a peak.* |
| 9. 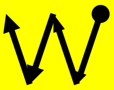 | | | *One shape looked like a letter.* |
| 10. 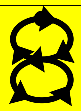 | | | *One shape looked like a number.* |
| ***11. Please name as many big cities in*** `[add name of your country]` ***or in the world as you can think of! You have 1 minute to do so*** | | | 1 point is awarded for each 3 cities<br>(8 points for 24 or more cities) |

`[note enumerated cities here]`

***“Please watch carefully and then try tapping the same pattern”.*** *(Speed 2 Hz= 2 beats per second). If the pattern is reproduced correctly, 2 points are awarded. If not, the pattern is given again by the administrator* ***(“I’ll tap the pattern again…. Please try again now”)****. If the pattern is now reproduced correctly, 1 point is awarded. B=both hands, L=left hand; R=right hand*

| | | | |
|---|---|---|---|
| *13. R-L-R-L-R-L-R-L* | | *14. L-R-R-L-R-R-L-R-R-L-R-R*<br>*(R-L-L-R-L-L etc. is also considered correct )* | |
| *15. B-L-B-R-B-L-B-R-B-L-B-R*<br>*(B-R-B-L etc. is also considered correct)* | | *16. B-L-R-B-R-L-B-L-R-B-R-L*<br>*(B-R-L-B-L-R etc. is also considered correct )* | |

| | |
|---|---|
| name: | |
| date of birth: | |
| date of testing: | |
| years of education: | |

| cognitive domains | score (max) | z-score |
|---|---|---|
| total (questions 1 to 16) | **(50)** | |
| semantic memory (questions 1 to 5) | **(10)** | |
| verbal memory (question 6) | **(8)** | |
| visual construction (questions 7 to 10) | **(8)** | |
| verbal fluency (question 11)<br>**Caution: The absolute number of cities named is decisive for the z-value!** | **(8)** | |
| visual memory (question 12) | **(8)** | |
| cognitive flexibility (questions 13 to 16) | **(8)** | |

## Hints regarding administration and scoring:

**Questions 1 to 4:**

Award 2 points each, if first question (e.g.: “*What is the name of the animal with the long trunk?”*) was answered correctly. Otherwise, give the additional hint, (e.g.: “*It's a very big animal that can 'trumpet' using its trunk”*). If the answer is now correct, award 1 point. In any case, name the animal you are looking for again: *"(Right.) The elephant was asked for"*.

**Question 5:**

Award 1 point for each correct answer.

**Question 6:**

For each animal that was remembered correctly (elephant, giraffe, zebra, polar bear), 2 points are given. For each animal that could NOT be remembered, give the three options. If the correct animal is selected, 1 point is awarded.

**Questions 7 to 10:**

If the shape is drawn correctly by the participant (shape can be clearly recognized, regardless if a mirror image is drawn or not) 2 points are awarded. If not, repeat the shape. If the shape is now drawn correctly, award 1 point.

**Question 11:**

Larger cities (>= 50 000 inhabitants) are counted. If the client chooses cities from only your county, point out that cities abroad are also counted. 1 point is awarded for each 3 cities (8 points for 24 or more cities). (Example: 7 cities → 2 points, 14 cities → 4 points etc.)

**Question 12:**

See question 6 above: For each shape that was remembered correctly without any hint, 2 points are awarded. Give hints for the remaining shapes that could not be remembered, (e.g.: *“one shape looked like a letter”*). If the correct shape then is drawn, one point is awarded.

**Questions 13 through 16:**

If a tapping pattern was given 0 points, do not administer the following tapping patterns. (Example: Client has been awarded 2 points for the first, 1 point for the second and no points for the third tapping pattern. The fourth tapping pattern will not be administered then).

**Interpretation of the total score:**

- < 38: Possible dementia syndrome
- < 41: Possible mild cognitive impairment (MCI). In this case, however, the „MCI-score“ (sum of the scores for "visual-spatial memory" and "cognitive flexibility") is more sensitive. Suspected MCI for MCI-scores < 11.

**Normative sample (n=136 participants without cognitive impairment) from Trapp et al. 2020, submitted)**

<table>
<tr><th rowspan="2"></th><th rowspan="2">Task</th><th>women</th><th>men</th></tr>
<tr><th colspan="2">mean (standard deviation)</th></tr>
<tr><td rowspan="6">age < 75</td><td>semantic memory</td><td colspan="2">9.78 (0.51)</td></tr>
<tr><td>verbal memory</td><td colspan="2">7.12 (0.77)</td></tr>
<tr><td>visual construction</td><td colspan="2">7.72 (0.64)</td></tr>
<tr><td>verbal fluency</td><td colspan="2">19.06 (6.55)</td></tr>
<tr><td>visual memory</td><td colspan="2">7.18 (1.21)</td></tr>
<tr><td>cognitive flexibility</td><td colspan="2">5.74 (1.66)</td></tr>
<tr><td rowspan="6">age >= 75</td><td>semantic memory</td><td colspan="2">9.75 (0.68)</td></tr>
<tr><td>verbal memory</td><td colspan="2">7.00 (0.90)</td></tr>
<tr><td>visual construction</td><td colspan="2">7.65 (0.57)</td></tr>
<tr><td>verbal fluency</td><td>17.62 (5.05)</td><td>20.32 (6.10)</td></tr>
<tr><td>visual memory</td><td colspan="2">6.87 (1.13)</td></tr>
<tr><td>cognitive flexibility</td><td colspan="2">5.23 (1.47)</td></tr>
</table>

“Years of education” (<=12 j vs. > 12 j) is without influence on the test results.
Gender differences were found for the verbal fluency task only (men >= 75j score higher than women >= 75j, $t_{(84)}=2.21$, p=.030).
