# Supplementary material for: Sensitivity and specificity of the Bamberg Dementia Screening Test’s (BDST) full and short versions: brief screening instruments for geriatric patients that are suitable for infectious environments
Source: BMC Med. 2021 Mar 5;19:65. doi: 10.1186/s12916-021-01927-4 (PMC7934397; doi:10.1186/s12916-021-01927-4)
Supplement: Supplementary file 2 — Additional file 2. BDST test form in German. [file 12916_2021_1927_MOESM2_ESM.pdf]

BDST

Zunächst stelle ich Ihnen einige Rätselfragen zu Tieren	Punkte Pr.	Punkte Abr.	*6. Nach welchen Tieren wurde gerade eben gefragt? (nur die ersten 6 Antworten werten)*
1. Wie heißt das Tier mit dem langen Rüssel?<br>*Es handelt sich um ein sehr großes Tier, das mit seinem Rüssel „trompeten“ kann*			*War Nilpferd, Elefant oder Nashorn dabei?*
2. Wie heißt das Tier mit dem sehr langen Hals?<br>*Das Tier lebt in Afrika und ist gelb-braun gemustert*			*War Leopard, Giraffe oder Papagei dabei?*
3. Wie heißt das Tier, nach dem ein schwarz-weiß gestreifter Fußgänger-Überweg benannt ist?<br>*Es handelt sich um ein Tier, das aussieht wie ein gestreiftes Pferd*			*War Zebra, Tiger oder Affe dabei?*
4. Wie heißt der Bär, der in sehr kalten Regionen lebt, in denen es Eis gibt?<br>*Setzen sie einfach die Worte „Bär“ und „Eis“ in der richtigen Reihenfolge zusammen*			*War Pinguin, Robbe oder Eisbär dabei?*
5. Vor welchem Tier haben die Menschen mehr Angst: Löwe oder Kaninchen?			
Warum? (gefährlich, Fleischfresser etc.)			

Bitte versuchen Sie, die folgenden Figuren in der Luft nachzuzeichnen *(2 Punkte falls erster, 1 Punkt, falls 2. Versuch richtig)*			***12. Bitte versuchen Sie jetzt noch einmal, alle Figuren von gerade eben aus dem Gedächtnis „in die Luft“ zu zeichnen***
7. 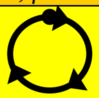			*Eine Figur war rund*
8. 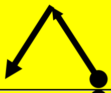			*Eine Figur sah aus wie eine Spitze*
9. 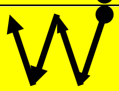			*Eine Figur sah aus wie ein Buchstabe*
10. 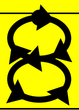			*Eine Figur sah aus wie eine Zahl*
11. Bitte zählen Sie mir jetzt so viele größere Städte in Deutschland oder in der Welt auf, wie Ihnen einfallen, Sie haben dafür 1 Minute Zeit“		Punkte (ganzzahlig, Vorkommastelle zählt) = Anzahl genannter Städte / 3, max 8	

Bitte versuchen Sie jetzt, genauso zu klopfen wie ich. *(Geschwindigkeit 2 Hz= 2 Schläge pro Sekunde. Falls nicht richtig wiederholt, noch einmal demonstrieren). 2 Punkte, falls richtig im ersten´, 1 Punkt falls richtig im zweiten Versuch, 0 Punkte sonst. B=beide Hände, L=linke Hand; R=rechte Hand*

13. L-R-L-R-L-R		*14. L-R-R-L-R-R-L-R-R-L-R-R (R-L-L-R-L-L etc. gilt auch)*	
15. B-L-B-R-B-L-B-R-B-L-B-R (B-R-B-L etc. gilt auch)		*16. B-L-R-B-R-L-B-L-R-B-R-L (B-R-L-B-L-R etc. gilt auch)*	

Name:	
Geburtsdatum:	
Testdatum:	
Ausbildungsjahre:	

Scores	Punktwert (Max)	z-Wert
Gesamt (Fragen 1-16)	**(50)**	
Semantik (Fragen 1-5)	**(10)**	
verbales Gedächtnis (Frage 6)	**(8)**	
Konstruktive Praxis (Fragen 7-10)	**(8)**	
Verbale Flüssigkeit (Frage 11)<br>**Achtung: Für den z-Wert ist die absolute Anzahl genannter Städte maßgeblich!**	**(8)**	
Visuell-räumliches Gedächtnis (Frage 12)	**(8)**	
Kognitive Flexibilität (Fragen 13-16)	**(8)**	

Bewertungs- und Vorgabehinweise:

Fragen 1 bis 4:

Jeweils 2 Punkte, falls erste Frage (z.B.: *Wie heißt das Tier mit dem langen Rüssel?*) richtig beantwortet wurde. Wurde die erste Frage nicht richtig beantwortet, „Hilfsfrage" (z.B.: *Es handelt sich um ein sehr großes Tier, das mit seinem Rüssel „trompeten" kann*) stellen. Falls das gesuchte Tier dann benannt wird, 1 Punkt vergeben. Das gesuchte Tier in jedem Fall noch einmal benennen: „(Richtig.) Der Elefant war gesucht".

Frage 5:

1 Punkt für jede richtige Antwort.

Frage 6:

2 Punkte für jedes im freien Abruf richtig genannte Tier (Elefant, Giraffe, Zebra, Eisbär). Bei nicht genannten Tieren die Alternativen vorgeben. Falls das richtige Tier dann erkannt wird, 1 Punkt vergeben.

Fragen 7 bis 10:

Jeweils 2 Punkte, wenn die Figur im ersten Versuch richtig reproduziert wurde. Falls dies nicht gelingt, die Figur noch einmal „vorzeichnen". Jeweils 1 Punkt, wenn die Figur im zweiten Versuch richtig reproduziert wurde.

Frage 11:

Es werden größere Städte (ab einer Einwohnerzahl von ca. 50 000) gewertet. Falls die Testperson ausschließlich Städte in Deutschland nennt, darauf hinweisen, dass auch Städte im Ausland gewertet werden. 1 Punkt pro drei genannter Städte, maximal 8 Punkte (für 24 oder mehr Städte) vergeben. (Bsp.: 7 genannte Städte → 2 Punkte, 14 genannte Städte → 4 Punkte etc.)

Frage 12:

Bewertung wie Frage 6: 2 Punkte für jede frei reproduzierte Figur. Für jede Figur, die nicht bzw. nicht richtig erinnert wurde, die jeweilige Hilfestellung (z.B.: *Eine Figur sah aus wie ein Buchstabe*) geben. Falls die Figur dann richtig wiedergegeben wird, 1 Punkt vergeben.

Fragen 13 bis 16:

Falls eine Aufgabe mit 0 Punkten bewertet wurde, die folgenden Aufgaben nicht mehr vorgeben. (Bsp.: Pat. hat auf das erste Klopfmuster 2 Punkte, auf das zweite 1 Punkt und auf das dritte Klopfmuster 0 Punkte erhalten. Das vierte Klopfmuster wird deshalb nicht mehr vorgegeben).

Interpretation des Gesamtscores:

- < 38: Verdacht auf dementielles Syndrom
- < 41: Verdacht auf leichte kognitive Störung (MCI), hier ist allerdings der „MCI-Score" (Summe der Scores für „visuell-räumliches Gedächtnis" und „kognitive Flexibilität") trennschärfer. Verdacht auf MCI bei MCI-Score < 11.

Normwerte (n=136 Kontrollen aus Trapp et al. 2020, submitted)

<table>
<tr><th rowspan="2"></th><th rowspan="2">Subtest</th><th>Frauen</th><th>Männer</th></tr>
<tr><th>M (STD)</th><th>M (STD)</th></tr>
<tr><td rowspan="6">Alter < 75</td><td>Semantisches Gedächtnis</td><td colspan="2">9.78 (0.51)</td></tr>
<tr><td>Verbales Gedächtnis</td><td colspan="2">7.12 (0.77)</td></tr>
<tr><td>Konstruktive Praxis</td><td colspan="2">7.72 (0.64)</td></tr>
<tr><td>Verbale Flüssigkeit</td><td colspan="2">19.06 (6.55)</td></tr>
<tr><td>Visuell-räumliches Gedächtnis</td><td colspan="2">7.18 (1.21)</td></tr>
<tr><td>Kognitive Flexibilität</td><td colspan="2">5.74 (1.66)</td></tr>
<tr><td rowspan="6">Alter >= 75</td><td>Semantisches Gedächtnis</td><td colspan="2">9.75 (0.68)</td></tr>
<tr><td>Verbales Gedächtnis</td><td colspan="2">7.00 (0.90)</td></tr>
<tr><td>Konstruktive Praxis</td><td colspan="2">7.65 (0.57)</td></tr>
<tr><td>Verbale Flüssigkeit</td><td>17.62 (5.05)</td><td>20.32 (6.10)</td></tr>
<tr><td>Visuell-räumliches Gedächtnis</td><td colspan="2">6.87 (1.13)</td></tr>
<tr><td>Kognitive Flexibilität</td><td colspan="2">5.23 (1.47)</td></tr>
</table>

Ausbildungsdauer (<=12 j vs. > 12 j) hat keinen Einfluss auf die Testwerte. Geschlechtsunterschiede ergeben sich nur für die verbale Flüssigkeitsaufgabe (Männer >= 75j sind besser als Frauen >= 75j, $t_{(84)}$=2.21, p=.030)
