# Supplementary material for: Sensitivity and specificity of the Bamberg Dementia Screening Test’s (BDST) full and short versions: brief screening instruments for geriatric patients that are suitable for infectious environments
Source: BMC Med. 2021 Mar 5;19:65. doi: 10.1186/s12916-021-01927-4 (PMC7934397; doi:10.1186/s12916-021-01927-4)
Supplement: Supplementary file 3 — Additional file 3. Correlations of the BDST scores with age, gender and years of education. [file 12916_2021_1927_MOESM3_ESM.docx]

|  | age | gender | years of education |
| --- | --- | --- | --- |
| BDST | -.20*** | -.05 | .10** |
| BDST_s_ | -.16*** | -.02 | .06* |
| BDST_us_ | -.16*** | -.06* | .10** |

Table: Correlations of the BDST scores with age, gender and years of education. ***: p< .0005, **: p<.01, *: p<.05, BDST: Bamberg Dementia Screening Test, BDSTs: Bamberg Dementia Screening Test, short form (sum score for the first and second subtest), BDSTus: Bamberg Dementia Screening Test, ultra short form (only score for the first subtest), MMST: Mini Mental State Examination.
